# Shotgun metagenomic and untargeted metabolomic analysis of gut microbiome associated with peripheral artery disease

**DOI:** 10.64898/2026.10.16.757428

**Published:** 2026-10-17

**Authors:** Sarbjeet Niraula, Jae Woong Jang, Jon Jung, Spencer L. Stirewalt, Megan Alagna, James Du, Christopher Staley, Patrick Seed, Stefan J. Green, Karen J. Ho

## Abstract

**Background:**

While the human gut microbiome is a potentially important and modifiable risk factor for cardiovascular diseases, the links between the gut microbiome and peripheral artery disease (PAD) and its clinical subgroups (claudication and chronic limb-threatening ischemia; CLTI) are not well understood.

**Methods:**

We used shotgun metagenomics and untargeted metabolomics to analyze the fecal samples of people with PAD and age- and sex-matched non-PAD controls collected in a cross-sectional manner. We used functional prediction pathways, a network analysis, and weighted gene co-expression network analysis of metagenomic, metabolomics, and clinical data to better understand PAD-associated dysbiosis.

**Results:**

Alpha and beta diversity were similar between the PAD (n=78) and non-PAD (n=20) groups. Differentially abundant species were dominated by those belonging to the Lachnospiraceae, Ruminococcaceae and Enterobacteriaceae families. Fecal butyrate levels were reduced between the non-PAD and PAD groups, but differences were not statistically significant. There was significantly reduced alpha diversity in the samples of the CLTI compared to claudication subgroup. Abundance of multiple butyrate producers were greater in the claudication group compared to CLTI. Network analysis revealed 5 meta-modules, each with specific associations to PAD presentation and metabolomic profiles, and species that are both top module hubs and differentially abundant between groups. Community interconnectedness appears to increase in claudication, followed by community disintegration in CLTI.

**Conclusions:**

This metagenomic-metabolomic analysis of the gut microbiome of individuals with PAD, highlights the importance of studying functional capacity, diversity, and connectivity between species. Microbial modules may allow an integrative view of the microbial ecology relevant to presence and progression of PAD.

